# Supplementary material for: The interactome of intact mitochondria by cross-linking mass spectrometry provides evidence for coexisting respiratory supercomplexes
Source: Mol Cell Proteomics. 2017 Dec 8;17(2):216–32. doi: 10.1074/mcp.RA117.000470 (PMC5795388; doi:10.1074/mcp.RA117.000470)
Supplement: Supplemental Data [file supp_RA117.000470_133922_0_supp_23978_fzffwf.zip › spectra_annotation/mito_DR_spectra_annotation/397-1-4-1-15-1.pdf]

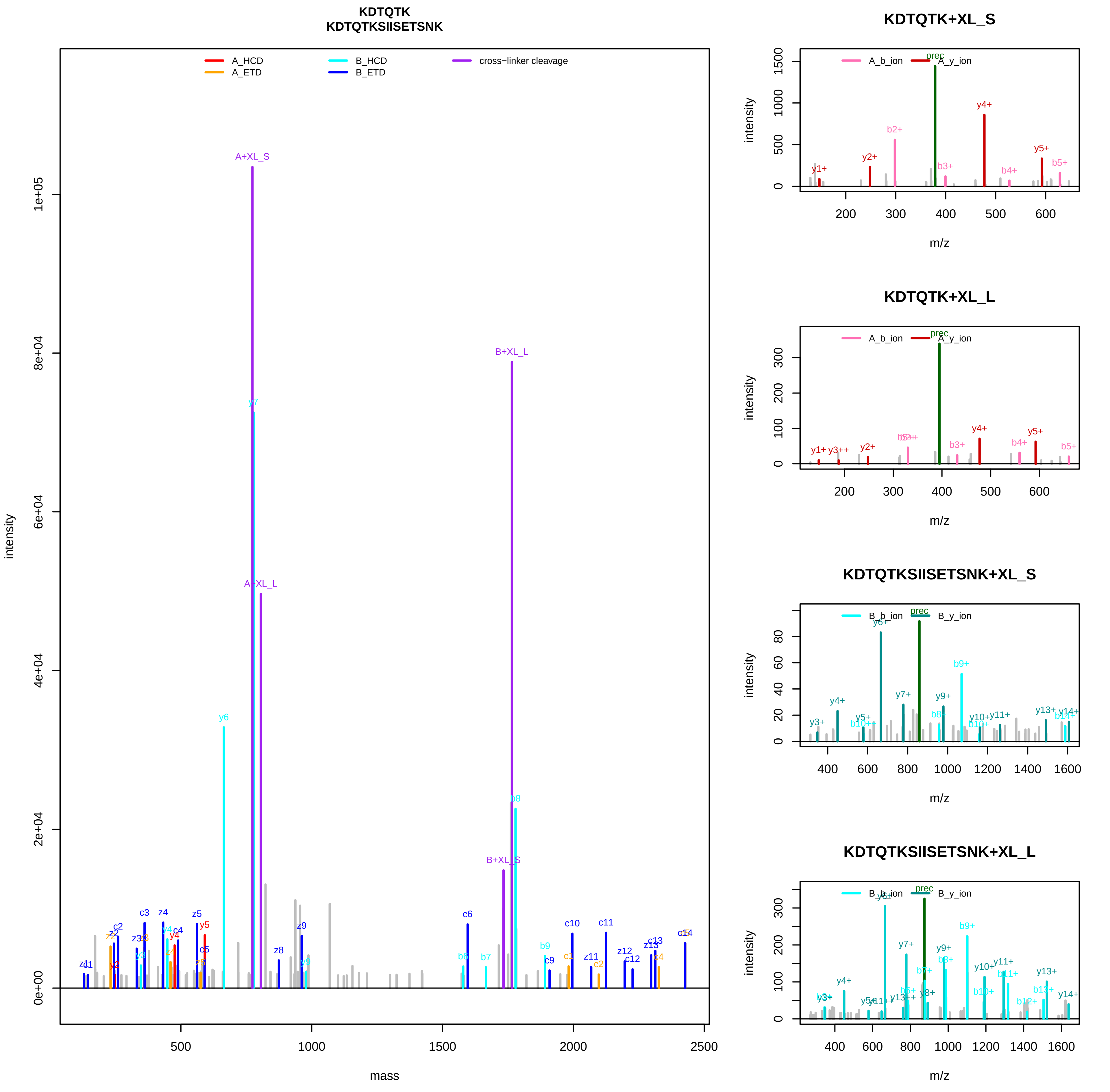

KDTQTK
KDTQTKSIISETSNK
A_HCD
A_ETD
B_HCD
B_ETD
cross−linker cleavage
intensity
mass
0e+00
2e+04
4e+04
6e+04
8e+04
1e+05
500
1000
1500
2000
2500
A+XL_S
A+XL_L
B+XL_L
B+XL_S
y7
y6
b8
b6
b7
b9
c1
c2
c3
c4
c5
c6
c9
c10
c11
c12
c13
c14
z1
z2
z3
z4
z5
z8
z9
z11
z12
z13
y2
y4
y5
y9
KDTQTK+XL_S
KDTQTK+XL_L
KDTQTKSIISETSNK+XL_S
KDTQTKSIISETSNK+XL_L
A_b_ion
A_y_ion
B_b_ion
B_y_ion
prec
m/z
y1+
y2+
y3++
y4+
y5+
b2+
b3+
b4+
b5+
y3+
y5+
y6+
y7+
y9+
y10+
y11+
y13+
y14+
b8+
b9+
b10+
b10++
b14+
